# Correction: IgG antibodies to SARS-CoV-2 in asymptomatic blood donors at two time points in Karachi

**DOI:** 10.1371/journal.pone.0315960

**Published:** 2024-12-12

**Authors:** Muhammad Hasan, Bushra Moiz, Shama Qaiser, Kiran Iqbal Masood, Zara Ghous, Areeba Hussain, Natasha Ali, J. Pedro Simas, Marc Veldhoen, Paula Alves, Syed Hani Abidi, Kulsoom Ghias, Erum Khan, Zahra Hasan

An additional affiliation is missing for the eighth author. J. Pedro Simas is also affiliated with the Universidade Católica Portuguesa, Católica Medical School, Católica Biomedical Research Centre, Lisboa, Portugal.
