# Supplementary figures and images for: IgG antibodies to SARS-CoV-2 in asymptomatic blood donors at two time points in Karachi
Source: PLoS One. 2022 Aug 24;17(8):e0271259. doi: 10.1371/journal.pone.0271259 (PMC9401161; doi:10.1371/journal.pone.0271259)

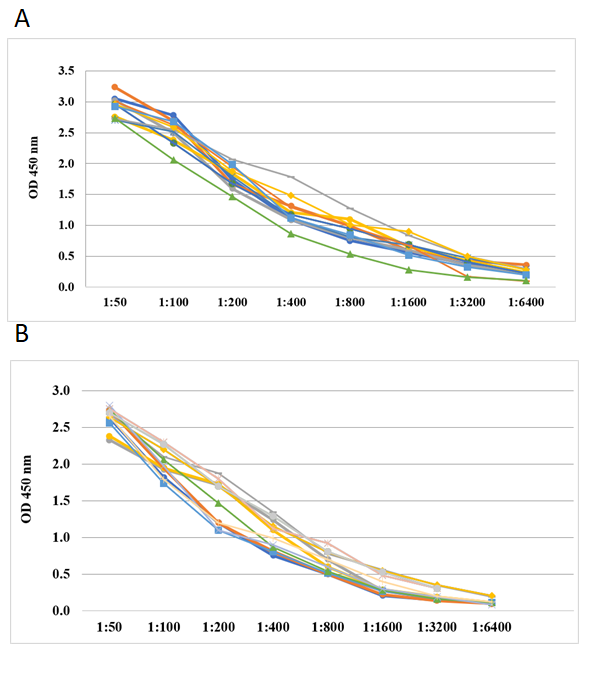

Supplement: S1 Fig — A positive serum pool containing high titers of anti convid19 antibodies was used to develop dilution curves. Serial 2-fold titrations from to 1/6400 were set up in each case. Graphs show an example of a dilution series for positive antibody pooled controls to either Spike protein (panel A) or RBD (panel B). A sigmoidal curve was obtained between a dilution of 50–3200 for both Spike and RBD proteins between OD 450 nm, 0.5–1.5. (TIF) [file pone.0271259.s001.tif]

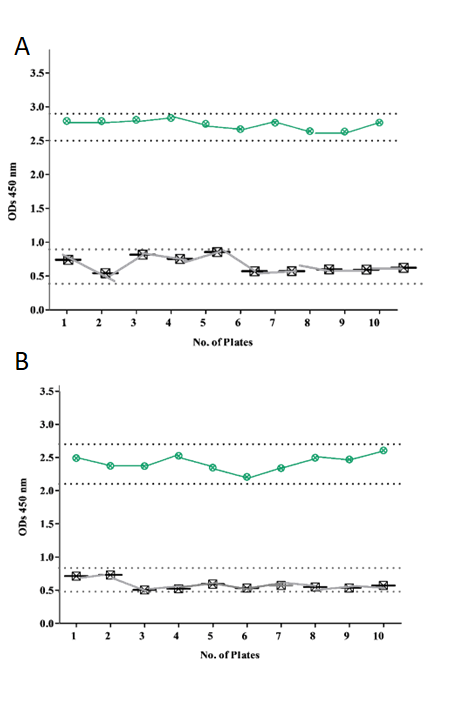

Supplement: S2 Fig — The variation between experiments for the higher and lower limits of dilution curves was determined for 10 consecutive assays for IgG antibodies to Spike (panel A), and RBD (panel B). The dotted lines indicate ± 2SD for each dilution. All other parameters are the same as in S1 Fig. (TIF) [file pone.0271259.s002.tif]

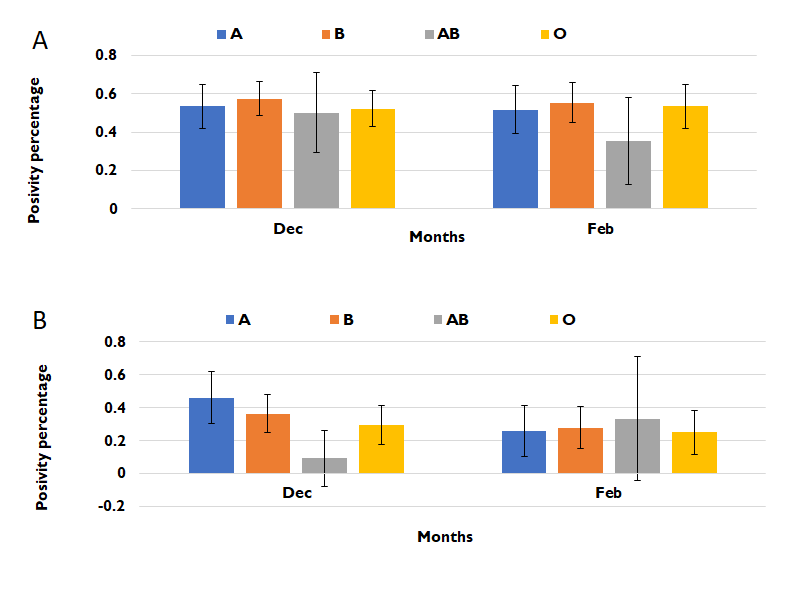

Supplement: S3 Fig — The graphs show the number of individuals tested in December 2021 and February 2022 with different blood groups. IgG positive to spike (A) and to RBD (B) are depicted in each case. Standard deviation of percentage positivity is shows as error bars. (TIF) [file pone.0271259.s003.tif]
