# Supplementary material for: IgG antibodies to SARS-CoV-2 in asymptomatic blood donors at two time points in Karachi
Source: PLoS One. 2022 Aug 24;17(8):e0271259. doi: 10.1371/journal.pone.0271259 (PMC9401161; doi:10.1371/journal.pone.0271259)
Supplement: S1 Table — (DOCX) [file pone.0271259.s004.docx]

**S Table 1. Gender- and blood type-distribution in various age bands**

| **Age group (years)** | **n (% of total)** | **Male**  **n (% of total)** | **Blood groups within age band** | | | |
| --- | --- | --- | --- | --- | --- | --- |
|  |  |  | A  n (%) | B  n (%) | AB  n (%) | O  n (%) |
| **17-25** | 224 (40.1) | 224(100) | 55(24.6) | 68(30.4) | 15(6.7) | 86(38.4) |
| **26-35** | 234 (41.9) | 232 (99.1) | 54(23.1) | 91(38.9) | 18(7.7) | 71 (30.3) |
| **36-45** | 85 (15.2) | 82 (96.4) | 19(22.4) | 35 (41.2) | 6(7.1) | 25(29.4) |
| **46-55** | 15 (2.7) | 15 (100) | 5(33.3) | 6 (40.0) | 0 | 4 (26.7) |
| **All** | 558 (100) | 553(99.1) | 133 (23.8) | 200 (35.8) | 39 (7.0) | 186 (33.3) |
