# Supplementary material for: IgG antibodies to SARS-CoV-2 in asymptomatic blood donors at two time points in Karachi
Source: PLoS One. 2022 Aug 24;17(8):e0271259. doi: 10.1371/journal.pone.0271259 (PMC9401161; doi:10.1371/journal.pone.0271259)
Supplement: Attachment — Submitted filename: PONE-reviewers_v2.docx [file pone.0271259.s006.docx]

PONE-D-21-30126

Increasing IgG antibodies to SARS-CoV-2 in asymptomatic blood donors through the pandemic in Karachi reflects exposure and immunity in the population

PLOS ONE


Journal requirements:

When submitting your revision, we need you to address these additional requirements.

1. Please ensure that your manuscript meets PLOS ONE's style requirements, including those for file naming. The PLOS ONE style templates can be found at

-Thank you. We have made the required style changes in the manuscript.

*2. Please provide additional details regarding participant consent. In the ethics statement in the Methods and online submission information, please ensure that you have specified what type you obtained (for instance, written or verbal, and if verbal, how it was documented and witnessed). If your study included minors, state whether you obtained consent from parents or guardians. If the need for consent was waived by the ethics committee, please include this information.*

-Written consent from each study participant. Only adults aged 18 years and over were included in the study.

*3. Thank you for stating the following financial disclosure:*

*“The study was supported by a grant from Provost’s Academic Priorities Fund, The Aga Khan University, Pakistan”*

*Please state what role the funders took in the study.  If the funders had no role, please state: ""The funders had no role in study design, data collection and analysis, decision to publish, or preparation of the manuscript.*

*If this statement is not correct you must amend it as needed.*

*Please include this amended Role of Funder statement in your cover letter; we will change the online submission form on your behalf.*

-Thank you for pointing this out. This has been added to the cover letter.

*4. Thank you for stating the following in the Funding Section of your manuscript:*

*“The study was supported by a grant from Provost’s Academic Priorities Fund, The Aga Khan University.”*

*We note that you have provided funding information that is not currently declared in your Funding Statement. However, funding information should not appear in the Acknowledgments section or other areas of your manuscript. We will only publish funding information present in the Funding Statement section of the online submission form.*

*Please remove any funding-related text from the manuscript and let us know how you would like to update your Funding Statement. Currently, your Funding Statement reads as follows:*

*“The study was supported by a grant from Provost’s Academic Priorities Fund, The Aga Khan University, Pakistan”*

*Please include your amended statements within your cover letter; we will change the online submission form on your behalf.*

- Thank you. This has been added to the cover letter.

*5. Please remove your figures from within your manuscript file, leaving only the individual TIFF/EPS image files, uploaded separately.  These will be automatically included in the -reviewers’ PDF.*

-Thank you. We will upload the image files separately.

Reviewers' comments:

5. Review Comments to the Author

Reviewer #1: This is a review of "Increasing IgG antibodies to SARS-CoV-2 in asymptomatic blood donors through the second COVID-19 wave in Karachi associated with exposure and immunity in the population" from Hasan et al, 2022, for consideration at PLOS ONE.

This paper could use numerous clarifications prior to publication, which I (hopefully) constructively suggest below.


\# Methods

*1. Line 71 states: "Any donor with a history of COVID-19 related symptoms or confirmed infection was excluded from enrollment as a blood donor." HOWEVER, the whole premise of the paper is that you have found high seropositivity among these blood donors. And, in the discussion, you discuss the likely levels of asymptomatic infection which explain this confusion. I would suggest that, around Line 71, you explicitly note that this exclusion criterion in your methods does not prevent those who were asymptomatically or paucisymptomatically infected from donation, which is key to the present study.*

- We thank the reviewer for this comment, the exclusion criteria has now been revised (line#80-81)

*2. Were the results adjusted for the sensitivity and specificity of the RBD and spike ELISAs? For that matter, what *were* the sensitivity and specificity of these assays? Were controls limited only to 100 known positive samples? Or were there also negative controls used? For instance, Figure 2 would be far better as seroprevalence estimates, with error bars, rather than raw sample counts which are not comparable between groups.*

*Methods for adjustment are discussed in the context of SARS-CoV-2 here in* [*https://elifesciences.org/articles/64206*](https://elifesciences.org/articles/64206) *and in general here* [*https://www.hindawi.com/journals/eri/2011/608719/*](https://www.hindawi.com/journals/eri/2011/608719/) *In general, the establishment of the ELISA cutoffs should be more clearly discussed, with positive and negative controls noted or referenced.*

- thank you for pointing this out. We have now included the calculation for sensitivity and specificity of the RBD and spike ELISA assays in the manuscript. The assay validation is explained more clearly. We used 45 COVID-19 convalescent sera as positive controls and 55 healthy sera from pre-pandemic period as negative controls.

- We have added confidence intervals (95%) to the results to explain the range of antibody responses investigated in each condition tested.

\# Results

*2. On Line 145, I suggest noting for the reader that 99.1% of donors were male, so the result about 99.7% of spike+ being men is not significant. Same with the 29.0±7.4 and 29.2±7.2. A reader who is skimming the paper may be surprised by these numbers.*

_ Thank you for pointing this out. We have re-written this line to avoid any confusion. *3. Do you have any estimate of how many of the samples might be RBD+ but spike-? On Line 294, the Discussion paragraph already notes that spike ELISAs are highly specific, so would you conclude that, perhaps, your RBD ELISA is relatively insensitive? If these numbers (RBD+, spike-) are not known, then this should be noted as a limitation.*

- None of the samples were spike negative but RBD positive. We have expanded our discussion of the results to describe our observation. The sensitivity and specificity of spike and RBD ELISAs are discussed. IgG to both these proteins were significantly correlated with each other.
- Further, it is noted that the dynamics of IgG to each of these proteins may differ in COVID-19.
   *4. "Our data showed an increasing frequency of seropositivity to spike and RBD in individuals who were between their second to fourth decades of life." Typically we would expect to see statistical tests of this claim, but I do not see them.*
- Thank you for this suggestion. We have re-analysed the data according to the age groups suggested by the reviewer. After doing so, we did not find any difference between IgG seropositivity to spike or RBD across the age groups. This has been corrected in the manuscript.


  # Data Availability

  -Data availability statement is corrected. The data has been depicted in tables and figures used for the manuscript. The full set will be available upon acceptance of the publication. Additional material can be provided upon request.


  # Small Suggestions

  *- Perhaps the numbers on Line 3 (Abstract) could be updated since it is now February 2022.*

  *- Refs 10 and 11 are the same paper, different title.*
- Thank you – this correction has been made.

*- L43-50 - place the literature review in dates or waves, but not "recent" or "previous". Years from now, it may be difficult to understand this paper without specific dates.*

*- BionaxNow spelling. (BinaxNow)*
 This correction has been made

*- A standard curve -[of] comprising a titration...*

Correction made

*- Were tested for the presence of -[for] IgG antibodies... awkward.*
correction made

*- Fig 1 — what do the symbols and shading mean?*

- "Similarly, though proportion of" -> "Similarly, the proportion of"

- "Overall, only 31.2% +[of] subjects having IgG to spike"

*- Supplementary Fig 1 - the horizontal axis doesn't line up with the data points. What are the horizontal error bar type things? Please clarify what the colors represent, or annotate them on the plots.*

- Thank you we have made this correction.

*Reviewer #2: The author can do a follow up spike and RBD antibodies in order to assess the concept of herd immunity in those positive cases. The work is good but is not novel as already one such study has been done in Karachi.*

-Thank you for this comment. We appreciate that there have been previous studies which have investigated the seroprevalence of antibodies to SARS-CoV-2. However, we believe it is of value to add to this body of literature in the context of different study populations and also locations across the country. Importantly, we discuss the value of using different antibody assays for identification of SARS-CoV-2 infections. Also, the possible significance of IgG to spike and RBD. Given that whilst spike is associated with protection to the virus, it is IgG to RBD which is directly correlated with neutralizing activity against SARS-CoV-2.

Reviewer #3: Authors attempted to assess SARS-CoV-2 seroprevalence in blood donors from Karachi, Pakistan, during the second COVID-19 pandemic wave.

There are several issues regarding this manuscript:

*1. The small sample size, significant gender imbalance and the distribution of age groups. For instance they have 4 participants in the age group 51-60 years and the age range for group 1 is 17-20: 4 years. For an unbiased observation authors should have grouped participants into different age categories, for example: 17-25; 26-35; 36-45; 46-55.*

-Thank you for this suggestion, we have re-analysed the data in the age categories as suggestion by the reviewer.

*2. It is not clear from the methods section the cut off for IgG positive test results. No information regarding the sensitivity and specificity of the test was provided.*
- We have added details regarding assay sensitivity and specificity and also cut-offs for IgG in the manuscript

*3. The manuscript needs an English review. Some terms are inadequate and some sentences are hard to understand. Few examples:*
- A review of the English in the manuscript as been conducted and corrections made.

4. Page 10: The current study performed December 2020 until February 2021 at the time
of the second COVID-19 wave shows a further rise (to 53%) in seropositivity in Karachi.
- correction made

5. Page 11: the secondary questionnaire was administered later…

- correction made.
