# Supplementary material for: IgG antibodies to SARS-CoV-2 in asymptomatic blood donors at two time points in Karachi
Source: PLoS One. 2022 Aug 24;17(8):e0271259. doi: 10.1371/journal.pone.0271259 (PMC9401161; doi:10.1371/journal.pone.0271259)
Supplement: Attachment — Submitted filename: Response to reviewers1.docx [file pone.0271259.s007.docx]

PONE-D-21-30126R1

Increasing IgG antibodies to SARS-CoV-2 in asymptomatic blood donors through the pandemic in Karachi reflects exposure and immunity in the population

PLOS ONE

Dear Dr. Hasan,

Thank you for submitting your manuscript to PLOS ONE. After careful consideration, we feel that it has merit but does not fully meet PLOS ONE’s publication criteria as it currently stands. Therefore, we invite you to submit a revised version of the manuscript that addresses the points raised during the review process.

==============================
ACADEMIC EDITOR:

One of the reviewers has some minor comments. Please address these before resubmitting
==============================

Reviewer 1

4. Have the authors made all data underlying the findings in their manuscript fully available?

The [PLOS Data policy](http://www.plosone.org/static/policies.action#sharing) requires authors to make all data underlying the findings described in their manuscript fully available without restriction, with rare exception (please refer to the Data Availability Statement in the manuscript PDF file). The data should be provided as part of the manuscript or its supporting information, or deposited to a public repository. For example, in addition to summary statistics, the data points behind means, medians and variance measures should be available. If there are restrictions on publicly sharing data—e.g. participant privacy or use of data from a third party—those must be specified.

Reviewer #1: No

>>

*Response: We thank the reviewer for pointing this out. We have now included all supporting data used for the manuscript as S1 Appendix.*

6. Review Comments to the Author

Please use the space provided to explain your answers to the questions above. You may also include additional comments for the author, including concerns about dual publication, research ethics, or publication ethics. (Please upload your review as an attachment if it exceeds 20,000 characters)

Reviewer #1: # Review 2

I have only a few more suggestions, which are small in nature. Overall, the manuscript is improved, and I find that the authors have addressed my comments, as well as the comments of the other reviewer. I thank the authors for their attentiveness, and recommend publication after some small adjustments are made:

- My biggest suggestion is to consider a change in title. Between the two time points, the authors do not show increasing seroprevalence. This fact makes the title potentially misleading. One alternative title might be:
"IgG antibodies to SARS-CoV-2 in asymptomatic blood donors at two time points in Karachi"
or something straightforward and clear like that.
>>>

*We thank the reviewer for their comments and have modified the title of the paper as suggested.*

- I suggest stating in the first part of the abstract that individuals with known COVID-19 or symptoms in the prior 4 weeks were excluded.

>>

*We thank the reviewer for their comments and have added this information in the Abstract*

- L168. "Hence" almost implies that the prior two percentages naturally lead to the third percentage. I suggest simply deleting "hence" to avoid this impression.

>>

*We have removed the work ‘Hence’ from L168*

- The figures appear low-resolution and blurry to me. Please check before final submission.

>>

*We thank the reviewer for their comments and have redone the TIF conversions for the files so that they are of better resolution*
